# Age-structured vectorial capacity reveals timing, not magnitude of within-mosquito dynamics is critical for arbovirus fitness assessment

**DOI:** 10.1186/s13071-020-04181-4

**Published:** 2020-06-15

**Authors:** E. Handly Mayton, A. Ryan Tramonte, Helen J. Wearing, Rebecca C. Christofferson

**Affiliations:** 1 Department of Pathobiological Sciences, School of Veterinary Medicine, Louisiana State University, Baton Rouge, LA USA; 2 Departments of Biology and Mathematics & Statistics, University of New Mexico, Albuquerque, NM USA; 3 Center for Computation and Technology, Louisiana State University, Baton Rouge, LA USA

**Keywords:** Extrinsic incubation period, EIP, Vector competence, *Aedes aegypti*, Vectorial capacity, Zika, Arbovirus, Mortality, Biting rate

## Abstract

**Background:**

Transmission dynamics of arboviruses like Zika virus are often evaluated by vector competence (the proportion of infectious vectors given exposure) and the extrinsic incubation period (EIP, the time it takes for a vector to become infectious), but vector age is another critical driver of transmission dynamics. Vectorial capacity (VC) is a measure of transmission potential of a vector-pathogen system, but how these three components, EIP, vector competence and vector age, affect VC in concert still needs study.

**Methods:**

The interaction of vector competence, EIP, and mosquito age at the time of infection acquisition (Age_acquisition_) was experimentally measured in an *Aedes aegypti-*ZIKV model system, as well as the age-dependence of probability of survival and the willingness to bite. An age-structured vectorial capacity framework (VC_age_) was then developed using both EIP_Min_ and EIP_Max_, defined as the time to first observed minimum proportion of transmitting mosquitoes and the time to observed maximum proportion of transmitting mosquitoes.

**Results:**

The within-mosquito dynamics of vector competence/EIP were not significant among treatments where mosquitoes were exposed at different ages. However, VC_age_ revealed: (i) age-dependence in vector-virus interactions is important for transmission success; (ii) lower vector competence but at shorter EIPs was sufficient for transmission perpetuation; and (iii) R_0_ may be overestimated by using non-age-structured VC.

**Conclusions:**

The results indicate that ultimately the temporal component of the virus-vector dynamics is most critical, especially when exposure occurred at advanced mosquito age. While our study is limited to a single virus-vector system, and a multitude of other factors affect both vector competence and mosquito mortality, our methods can be extrapolated to these other scenarios. Results indicate that how ‘highly’ or ‘negligibly’ competent vectors are categorized may need adjustment.
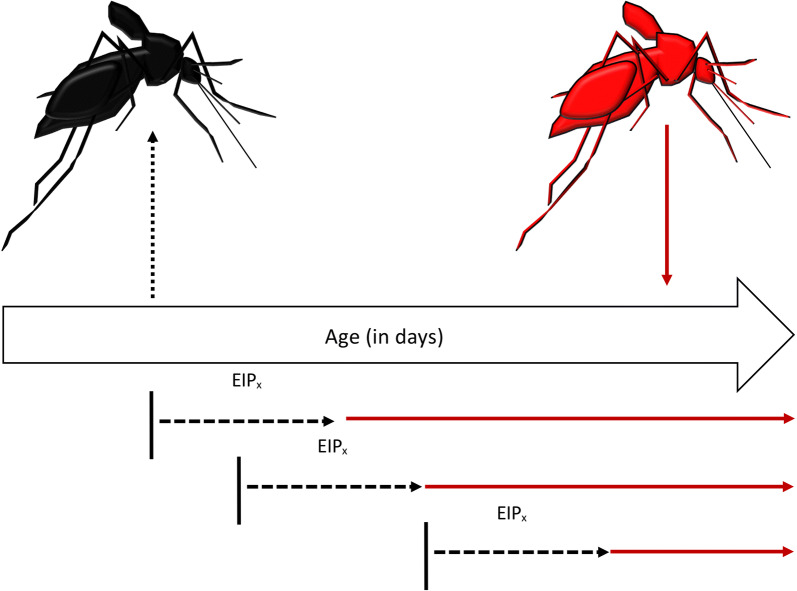

## Background

The transmission dynamics of arboviruses such as Zika virus (ZIKV) are evaluated over several characteristics, namely vector competence and the extrinsic incubation period (EIP). *Aedes aegypti* is the primary vector of ZIKV and several studies have evaluated its competence to transmit the virus [1–4]. Vector competence is the ability of a mosquito to acquire and ultimately transmit a virus [5, 6]. The time it takes for this process to occur is referred to as the extrinsic incubation period (EIP) [7]. Vector competence and EIP are interrelated measures of the proportion of vectors that become infectious given exposure and the time it takes for a vector to become infectious given exposure, respectively [6, 8]. Thus, EIP can be described as the temporality of vector competence and has been used to evaluate the relative fitness of arbovirus systems [6, 8, 9]. In addition, EIP and vector competence are influenced by many parameters including vector species, mosquito-immune system, microbiota fauna, discrete populations within species, and environmental factors [2, 3, 5, 10–15].

Indeed, changes in arbovirus fitness and thus transmission dynamics have been predicated on altered vector competence, especially as a critical component of vectorial capacity [6, 16–24]. The composite of vector competence and EIP into a single, dynamic measure allows for a more comprehensive understanding of this process [5, 10, 25–27]. Not all mosquitoes that are exposed will be able to transmit (vector competence) and the time it takes for those mosquitoes that will transmit is not a constant (EIP), and so understanding this composite over several days post-infection is critical [6, 28].

Vectorial capacity (VC) was derived as a measure of transmission potential of a vector-borne pathogen by a competent vector, and incorporates both vector competence and EIP [6, 7, 17, 18, 25, 29]. VC is the vector-centric component of the basic reproduction number (R_0_), and VC represents the number of secondary cases resulting from the introduction of a single infectious human individual per infectious day of that human index case [30, 31]. VC is given by:

1 $$VC = \frac{{ma^{2} bp^{N} }}{{ - { \ln }\left( p \right)}}$$

where ‘m’ is the density of mosquitoes relative to humans, ‘a’ is the biting rate, ‘b’ is the vector competence of the mosquito for a particular virus, ‘N’ is the extrinsic incubation period, and ‘p’ is the daily probability of mosquito survival [6]. And R_0_ is given by:

2 $$R_{0} = \frac{c}{r}*VC$$

where ‘r’ is the human recovery rate, or the reciprocal of the average human infectious period, and ‘c’ is the probability of transmission from the human to the vector given contact. R_0_ is a threshold parameter, whereby an R_0_ value < 1 is not expected to sustain transmission and an R_0_ value ≥ 1 is likely to result in an outbreak [11, 32].

VC uses an average daily probability of survival to calculate the probability of a mosquito living through the EIP (p^N^) and p^N^/(− ln(p) represents the expected infectious days given N and p [33]. It is intuitive how mortality could impact transmission, but most studies address mortality based on a constant age at which the mosquito acquires the infection [9, 10, 12, 34, 35]. Mathematical modeling studies have addressed age structure with respect to EIP or bite-structure, but no studies have empirically investigated all three processes simultaneously [9, 36]. Thus, herein, we experimentally test the hypothesis that transmission dynamics of arboviruses are as or more affected by time as a function of age *versus* time as a function of days post-infection. We further develop an age-structured vectorial capacity equation (VC_age_) to quantify these potential effects.

## Methods

We first wanted to determine if age and/or prior blood meals affected the within-mosquito viral dynamics, as well as various life traits of the mosquito. To that end, we designated three treatment groups: YOUNG, OLDER and S.OLDER. The YOUNG group was offered an infectious blood meal at approximately 5 days post-emergence (dpe). The OLDER group was offered a non-infectious blood meal at 5 dpe and then an infectious blood meal 1 week later (12 dpe). The S.OLDER group was not offered a prior, non-infectious blood meal, but a single infectious blood meal at approximately 12 dpe (to match the OLDER timing). S refers to sugar, which was done in order to distinguish the absence of a blood meal at 5 dpe. All non-infectious blood meals are referred to as “mock” blood meals, as they contain non-infectious cell culture media in place of infectious media.

### Virus and mosquitoes

ZIKV strain PRVABC59 (Asian lineage), originally isolated from a human patient in Puerto Rico in 2015, was provided by Dr Barbara Johnson at the USA Centers for Disease Control and Prevention (GenBank: KU501215). The viral stock was passaged three times in Vero cells before passage on Vero cells for mosquito exposure. Supernatant was collected 3 days post-inoculation and titer determined as previously described [37]. The titer of ZIKV was verified by qRT-PCR at approximately 8 × 10^7^ plaque forming units (pfu)/ml, matched across all exposure experiments. Virus used for mosquito exposure was never frozen, as this has been shown to negatively affect vector competence when compared to freshly cultured virus [38, 39]. Colony *Aedes aegypti* (Rockefeller) were provided by Dr Daniel Swale of the LSU Entomology Department, Baton Rouge, Louisiana, USA. To isolate the effect(s) of age, mosquitoes were maintained at constant conditions with 16:8 h light:dark periods, approximately 80% humidity (monitored using a digital RH monitor), and at 28 °C constant temperature. Mosquitoes were supplied with a 10% sucrose water solution after emergence *via* soaked cotton pledgets, which were replenished every 24 h. Sucrose solution was removed 24 h before experiments and was again provided after blood feeding/mock starvation.

### Blood-feeding and oral exposure of *Ae. aegypti*

Infectious and non-infectious blood meals were prepared with ZIKV-infected cell culture supernatant and non-infected cell culture supernatant, respectively. Whole bovine blood in Alsever’s solution (Hemostat Labs, Dixon, CA, USA) was used in a 2:1 blood to supernatant ratio [28]. Mosquitoes were fed *via* the Hemotek membrane feeding system (Hemotek, Blackburn, UK) for 45 min, after which mosquitoes were cold anesthetized and blood-fed females were placed into clean cartons until further treatment. Because starvation and cold anesthetization could affect mortality rates, every treatment was subjected to the same schedule of each of these conditions. For example, all groups were starved 24 h prior to days 5 and 12 post-emergence, regardless of whether that group would receive a blood meal. In addition, not only were blood-fed mosquitoes cold anesthetized and moved to a new carton, but other groups that did not feed on that schedule were treated the same in order to mimic these conditions and control for these factors. The experimental design is depicted in Additional file 1: Figure S1.

### Within-mosquito kinetics

In order to determine if the timing of the infectious blood meal affected the within-mosquito viral kinetics, mosquitoes were sampled at 5, 8 and 11 days post-infection (dpi) to test for infection and dissemination across all groups (sample sizes are provided in Additional file 1: Table S1). Mosquito legs and bodies were placed into separate tubes containing 900 μl BA-1 diluent media and BBs. Samples were then homogenized twice at 25 Hz for 3 min using a Qiagen Tissuelyzer (Qiagen, Hilden, Germany). RNA was extracted (5× MagMax 96 Viral RNA Isolation Kit; Applied Biosystems, Foster City, CA, USA) and tested for the presence of viral RNA *via* qRT-PCR (SuperScript III One-Step RT-PCR System; Invitrogen, Carlsbad, CA, USA) as in [40]. Each treatment was repeated a total of three times and data was averaged over these replicates. Differences among the treatment groups for infection and dissemination rates were tested by a chi-square test for multiple proportions on 5, 8 and 11 dpi.

### Transmission assay

To directly assess transmission potential, 20 mosquitoes per treatment were force-salivated. Briefly, ZIKV-exposed mosquitoes were immobilized on ice before removing legs and wings. Mosquitoes were then placed on double-sided tape, and the proboscis of each mosquito was placed into a pipette tip containing 35 μl FBS with 3 mmol/l ATP for 30 min, as previously described [41]. Contents of the pipette tip were ejected into 100 μl BA-1 diluent and stored at − 80 °C before testing (see below).

Mosquitoes from the two OLDER groups were sampled at 5, 8 and 11 dpi, as well as 16 dpi to represent the end of the mortality study (28 dpe). In order to characterize the EIP across the mosquito’s lifetime, mosquitoes in group YOUNG were sampled at 5, 8 and 11 dpi, as well as additional days post-infection in order to match the age at which the older groups were sampled. Thus, by adding 7 to the time points in the older groups, we additionally sampled the YOUNG group at 12, 15 and 18 dpi, as well as 23 dpi which corresponds to the oldest time point of 28 days old. Samples were tested for the presence of viral RNA in saliva using the techniques described above [40, 41]. Differences among the treatment groups’ transmission rates were tested by Chi-square test for multiple proportions at each sampling timepoint.

### Mortality study

Mortality studies were performed for the same three treatments (YOUNG, OLDER, and S.OLDER). We added additional mock blood meal controls (that is, accounting for any infectious blood meal-associated alteration of mortality) where a mock blood meal was used in place of infectious blood meals. The three controls were: (i) a mock blood meal at 5 dpe (M.Y) to correspond to the YOUNG treatment; (ii) a mock blood meal at 5 dpe, followed by another mock blood meal at 12 dpe (M.M) to correspond to the OLDER two blood meal treatment; and (iii) a mock blood meal at 12 dpe (S.M) to correspond to the S.OLDER, one blood meal treatment. An additional negative control treatment was performed where the mosquitoes were never blood-fed (S). All treatments were cold anesthetized at 5 and 12 dpe, regardless of whether they were offered a blood meal so that all mosquitoes experienced the same treatment, and mosquito density per carton was kept relatively constant with an average of 47 mosquitoes/carton (range 36–58). Each mortality treatment was repeated a total of three times and data are averages of the three replicates.

Cartons were checked daily and, when present, dead mosquitoes were counted and removed up to 28 dpe (approximately 1 month), as this has been shown to be the upper limit of field survival of *Ae. aegypti* and is similar to the range used in Tesla et al. [13, 41]. Only mosquitoes that took all offered blood meals were included in the mortality analyses.

To test for differences in mortality rates among treatments, Kaplan-Meier survival analyses were conducted and the average time to death (TTD) was estimated. Daily mortality estimates relative to age were then predicted using best fits in R (version 3.5.2) [42].

### Determining age-structured willingness to probe

One of the most influential parameters for determining transmission dynamics of vector-borne pathogens is the biting rate (because it impacts vector-host transmission in both directions). Biting rate is sometimes parameterized as the reciprocal of the number of days between feeds [43]. This assumes that the waiting time between bites is exponentially distributed, and we make this assumption for three biting rates: 0.5 (once every two days); 1 (once a day); and 2 (twice a day) [44]. However, we wanted to determine if the willingness of the mosquito to probe was affected by (i) timing of infectious blood meal and/or (ii) age of the mosquito [36]. While mosquito biting is a function of many factors, it has been shown that heat cues are sufficient to initiate host-seeking behaviors [45]. We use this to determine the willingness or probability of a mosquito probing, which can lead to transmission [46–48].

Ten to twelve mosquitoes per each ZIKV-infected treatment (YOUNG, OLDER and S.OLDER) were placed in individual, clear plastic canisters (Bioquip, Rancho Dominquez, CA, USA) 24 h before being provided a blood meal *via* a membrane feeder using 1 ml discs with 800 μl of blood (Hemotek, Blackburn, UK). This was done at the same dpi schedule as the vector competence studies above. Willingness to bite was assessed using a two-tiered approach by a single observer to control for observation bias. First, mosquitoes were observed through the clear canister for their general position in the canister and secondly, the disc was removed to determine if they were on or near the mesh at the top of the canister. In all cases, these two methods of observation matched. That is, if a mosquito was observed to be at the blood meal prior to disc removal (looking through the canister), it did not move to the bottom of the canister upon disc removal.

This observation was performed at 1, 20 and 45 min post-placement of the disc and the disc was replaced between observation time points. Thus, a mosquito was assessed as “landed” and recorded as “1” if the female was at the top of the canister at any of the observation times. The female was otherwise classified as “not landed” and coded as “0” if she was at the bottom of the canister for all three observation times. We then calculated the probability of biting (Z) as a function of age. Z(age) was determined by fitting the proportion of mosquitoes that landed or fed at least once a day using a self-starting non-linear least squares regression:

3 $$Z\left( {age} \right) = s + \left( {g - s} \right){ \exp }( - \exp \left( h \right)*\left( { - age} \right))$$

where ‘s’ is the asymptote, ‘g’ is the zero-response parameter, and ‘h’ is the logarithmic rate constant. Comparison of probing differences among treatments was assessed both per day post-infection as well as per mosquito age by Kruskal-Wallis test.

### Age-structured vectorial capacity and R_0_

**Fig. 1:**
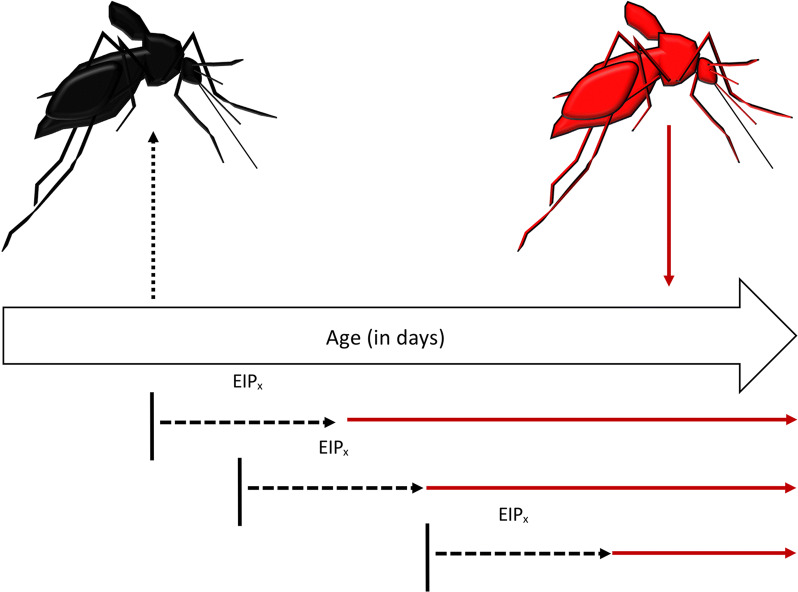
Vector age at time of infection acquisition determines transmission opportunity. Uninfected mosquitoes (black, left) acquire an infection at Age_acquisition_ (black vertical lines) and after a certain EIP (black dashed lines), a proportion will become infectious (red, right), but for a certain period of time (red horizontal lines) that is dependent on Age_acquisition_

We re-formulated the vectorial capacity equation to estimate VC as a function of the age at which the mosquito acquires an infectious blood meal, redefining the parameters with respect to age at the time of acquisition of infection. We define Age_acquisition_ as the age at which a mosquito acquires an infection (the day of taking the infectious blood meal) and Age_transmission_ as the age at which the mosquito subsequently transmits (Fig. 1):

4 $$Age_{transmission} = Age_{acquisition} + EIP$$

In VC_age_, ‘m’ is still the mosquito-to-human density and, for illustrative purposes, held constant here at 1. The parameters z_acquisition_ and z_transmission_ are the probability of a mosquito biting at age of acquisition and age at time of transmission, respectively. The traditional calculation of p^N^ represents the probability of a mosquito living through a fixed number of days N, the EIP, and p^N^/(− ln(p)) represents the expected infectious days given N and p [33]. In the context of an age-dependent vectorial capacity framework, we can calculate a more precise probability of surviving based on the day the mosquito obtained an infection and the cumulative survival probability to Age_transmission_ where the cumulative probability of living through the EIP given Age_acquisition_ is given by:

5 $$p\left( {surviving \, to \, Age_{transmission} } \right) = \mathop \prod \limits_{j = 1}^{{Age_{transmission} }} p_{j}$$

where ‘p_j_’ is the probability of daily survival on day *j* post emergence. We further estimate the infectious period (L) in an age-structured way by numerically deriving L:

6 $$L = TTD - Age_{transmission}$$

where TTD is the average time-to-death derived from the experimental mortality study.

Using the data from our experimental studies, we calculated Age_transmission_ for two scenarios: EIP_min_ and EIP_max_ from our observed transmission data [49], and calculated VC_age_ as:

7 $$VC_{age} = mb\left( {z_{acquisition} a} \right)\left( {z_{transmission} a} \right)\left( {\mathop \prod \limits_{j = 1}^{{age_{transmission} }} p_{j} } \right)L$$

VC is a component of R_0_, which also includes the average infectious period of the human and the probability of transmission from the human to the vector. For illustrative purposes, we calculated R_0_ using both the traditional calculation of VC (Eq. 1), as well as VC_age_. We made the following assumptions: mosquito density is held constant at m = 1; the average infectious period of the human is 5 days (r^−1^) [50]; and the average probability of transmission from human to vector is parameterized experimentally as the average proportion of mosquitoes that develop a midgut infection given exposure (c). From these data, we can calculate the value of VC needed to push R_0_ above this threshold. We use this value as a “threshold” to compare VC_age_ across treatment groups and with the traditional VC measures.

All statistics and subsequent graphics were performed and generated using R version 3.5.2 [42]. All packages used are provided in Additional file 1: Text S1. All functions and function parameters used to fit the data and obtain age-dependent distributions of these parameters are given in Additional file 1: Table S2. Goodness-of-fit was assessed either through AIC (for non-linear models) or *R*^2^ for linear models.

## Results

### Within-mosquito dynamics are more affected by time as a function of age *versus* days post-infection

**Fig. 2:**
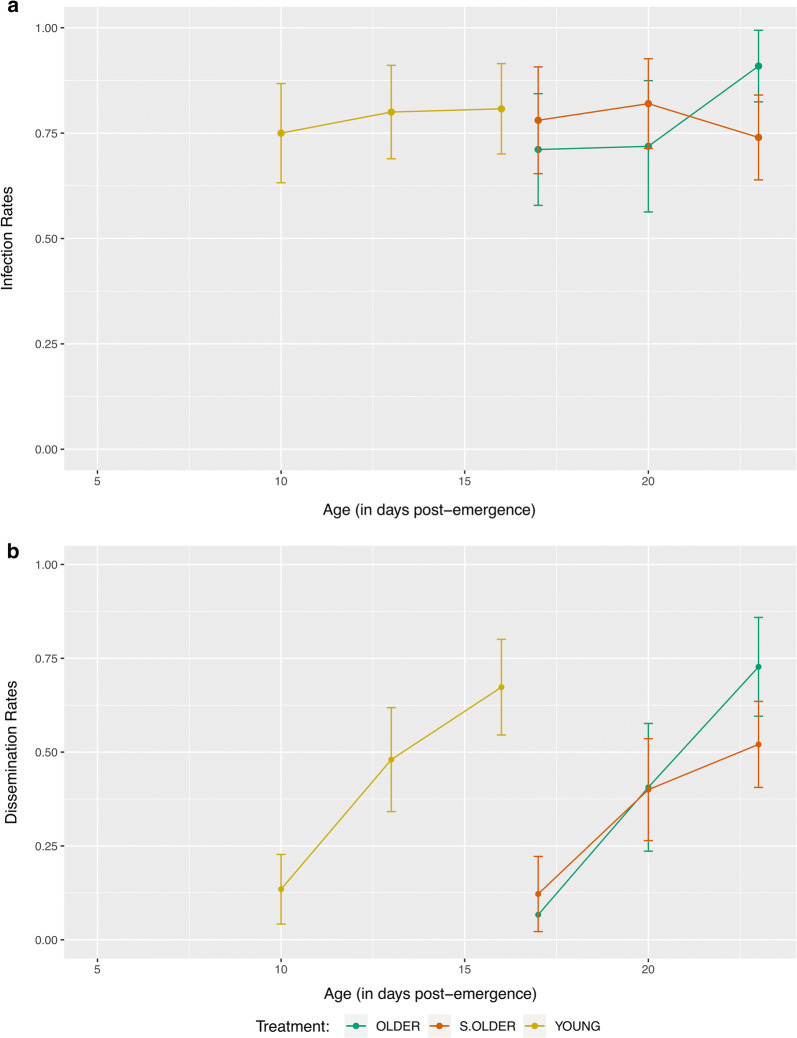
Infection and dissemination rates in the context of mosquito age. Despite no significant effect of treatment on infection and dissemination rates when assessed over days post-infection, Age_acquisition_ has an obvious impact on the timing of these processes

We first wanted to determine if the age at which a mosquito is offered an infectious blood meal and acquires the ZIKV infection (Age_acquisition_) impacted the within-vector kinetics of the mosquito. To do this, we looked at infection, dissemination, and transmission of the three treatments in the context of days post-infection. We found no significant difference in the infection (*P* > 0.05) or dissemination rates (*P* > 0.05) across all treatments (Additional file 1: Table S1). However, when direct age comparisons are made, the effect of Age_acquisition_ on infection becomes obvious (Fig. 2). The average rate of infection of mosquitoes given exposure was 78.2% across all treatments and days post-infection (95% confidence interval: 74.3–82.0%).

When we evaluated the proportion transmitting in terms of days post-exposure (5, 8 and 11 only) none of the treatments were transmitting on days 5 or 8 dpi. On day 11, the transmission rates were 5%, 10% and 15% in groups OLDER, S.OLDER and YOUNG, respectively. These differences were not statistically significant using the Chi-square test for proportions (*P* > 0.5) (Additional file 1: Table S1).

However, when we investigated mosquitoes from group YOUNG at time points that age-matched OLDER and S.OLDER treatments (Table 1), the YOUNG group achieved a maximum transmission of 45% at 28 days old (23 dpi) *versus* only 15% for the S.OLDER group and 10% for the OLDER group at 28 days old (16 dpi).

**Table 1:** Transmission rates for each day post-infection (dpi) and corresponding mosquito age for each of the three treatment groups

Treatment	dpi (day post-infection)	Age (day post-emergence)	% Transmission (*n *=20)
YOUNG	5	10	0
	8	13	0
	11	16	15
	12	17	15
	15	20	10
	18	23	35
	23	28	45
OLDER	5	17	0
	8	20	0
	11	23	5
	16	28	10
S.OLDER	5	17	0
	8	20	0
	11	23	10
	16	28	15

We define the EIP_max_ as the earliest EIP (days post-infection) where the maximum proportion of mosquitoes are transmitting, and we define the EIP_min_ as the earliest EIP where the proportion of mosquitoes transmitting is minimal, but greater than 0. EIP values at different vector competence levels have been used to evaluate the vectorial capacity for malaria [49]. For the YOUNG group, EIP_max_ was 23 dpi at 45%, and EIP_min_ was 11 dpi at 15%. For the OLDER group, EIP_max_ was 16 dpi at 10% and EIP_min_ was 11 dpi at 5%. For the S.OLDER group, EIP_max_ was 16 dpi at 15% and the EIP_min_ was 11 dpi at 10%.

### Mortality of *Aedes aegypti* is modestly affected by timing of infectious blood meal

When each infectious treatment was compared to a matching non-infectious control group, the only significant difference in average time to death (TTD) was between the S.OLDER and S.M treatments, with an estimated difference in TTD of two days (Additional file 1: Table S3). Of interest, the non-blood-fed sugar-only controls died significantly faster than any of the blood-fed treatments with an average TTD of 19.6 days (Additional file 1: Table S3), which has been previously shown [51].

**Fig. 3:**
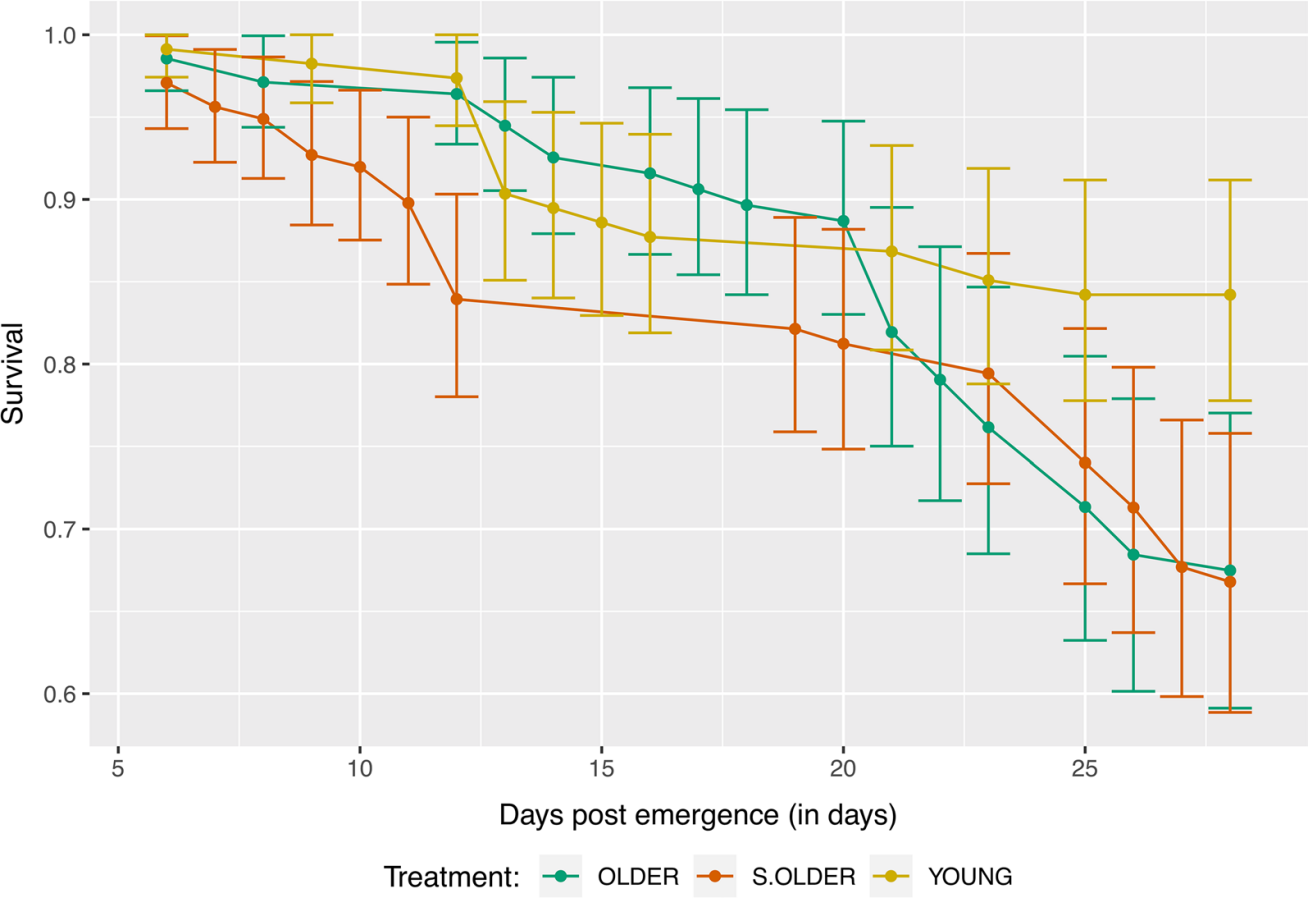
Survival curves of female *Ae. aegypti* by treatment. Each line represents the combined data from three replicates per treatment: YOUNG (gold line), OLDER (green line), and sugar-OLDER (S.OLDER, red line). Average time to death of treatment, YOUNG was significantly, but modestly, longer than treatments OLDER and S.OLDER

Pairwise comparisons of the ZIKV-exposed treatments determined that group YOUNG had a significantly longer average time to death (TTD) when compared to groups OLDER and S.OLDER, though this difference was modest (0.6 and 1.4 days, respectively) (Fig. 3). The TTD for the YOUNG group was 25.9 days, 25.3 days for the OLDER group, and 24.5 days for the S.OLDER group. This corresponds to average daily survival probabilities of 0.962, 0.961 and 0.960 for the YOUNG, OLDER and S.OLDER groups, respectively. Predicted daily survival rates were generated for the YOUNG group using a non-linear fit and a linear model was fit to the OLDER and S.OLDER groups. The parameters of these models and goodness-of-fit assessments are given in Additional file 1: Table S2, and the observed and predicted values are shown in Additional file 1: Figure S2 for all three treatment groups. Additional comparisons were made among the treatment and control groups, detailed in Additional file 1: Text S2, Figure S3 and Table S3.

### Age-dependence of willingness to probe

**Fig. 4:**
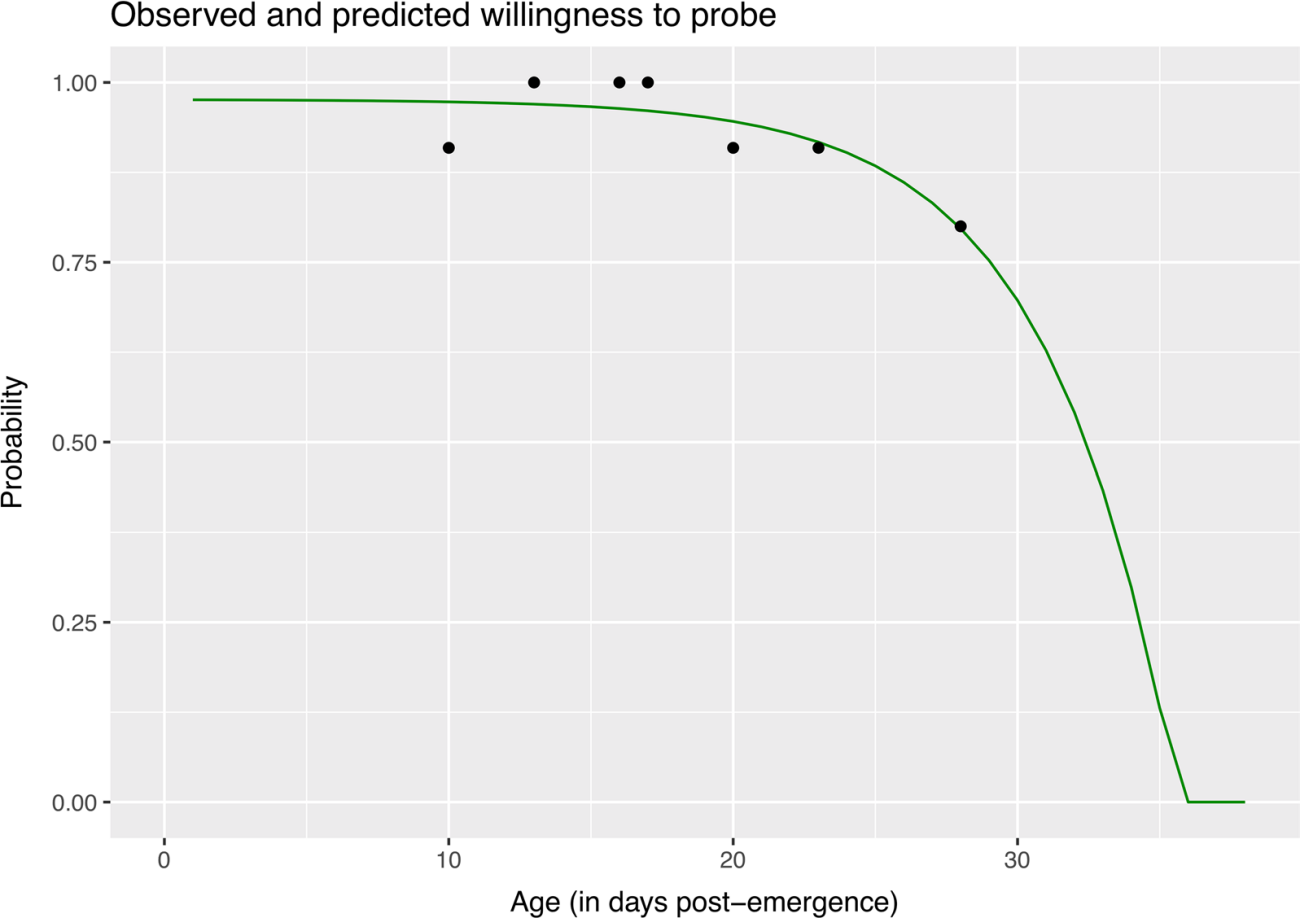
Observed and predicted probabilities of daily biting. The observed daily biting frequencies (dots) from the laboratory experiments and the fitted daily predictions (green curve)

There was no significant difference in the willingness to probe based on treatment (YOUNG, OLDER and S.OLDER) at each day post-infection *via* the Kruskal-Wallis test, but there was a significant effect of age (*P* < 0.05). We then were able to fit a daily probability of probing based on age (Fig. 4).

### VC_age_ framework for assessing transmission as a function of age and effect on R_0_

To calculate VC_age_, we used treatment-group specific average TTD and predicted daily probabilities of survival (Additional file 1: Figure S4) and the overall daily prediction of willingness to bite in Fig. 4. Since biting rate (the number of bites per day) is a field-derived parameter, we calculated VC_age_ over three biting rates: 0.5 (once every 2 days), 1 (once a day), and 2 (twice a day) [28]. For comparison, we calculated the traditional VC using the average life-dependent traits determined experimentally above, EIP_min_ and EIP_max_, and the biting rates referenced above. These VC values are given in Table 2, along with corresponding values for R_0_. In the absence of published data regarding the probability of human to mosquito ZIKV transmission success, we used the average infection rate from our experimental data (78.2%) to parameterize human to mosquito transmission, recognizing that this model system used a high dose of ZIKV titer, although such titers in individuals are not unheard of [52, 53].

**Table 2:** Traditional VC calculations for EIP_min_ and EIP_max_ for each treatment group (calculated according to Eq. 1) and corresponding R_0_ (according to Eq. 2)

Treatment	EIP	Biting rate	VC	R_0_
YOUNG	EIP_max_	2	19.06	74.52
		1	4.77	18.65
		0.5	1.19	4.65
	EIP_min_	2	10.11	39.53
		1	2.53	9.89
		0.5	0.632	2.47
OLDER	EIP_max_	2	4.03	15.76
		1	1.01	3.95
		0.5	0.25	0.978
	EIP_min_	2	6.49	26.98
		1	1.62	6.33
		0.5	0.41	1.60
S.OLDER	EIP_max_	2	7.65	29.91
		1	1.91	7.47
		0.5	0.48	1.88
	EIP_min_	2	6.25	24.44
		1	1.56	6.10
		0.5	0.39	1.52

### YOUNG group

**Fig. 5:**
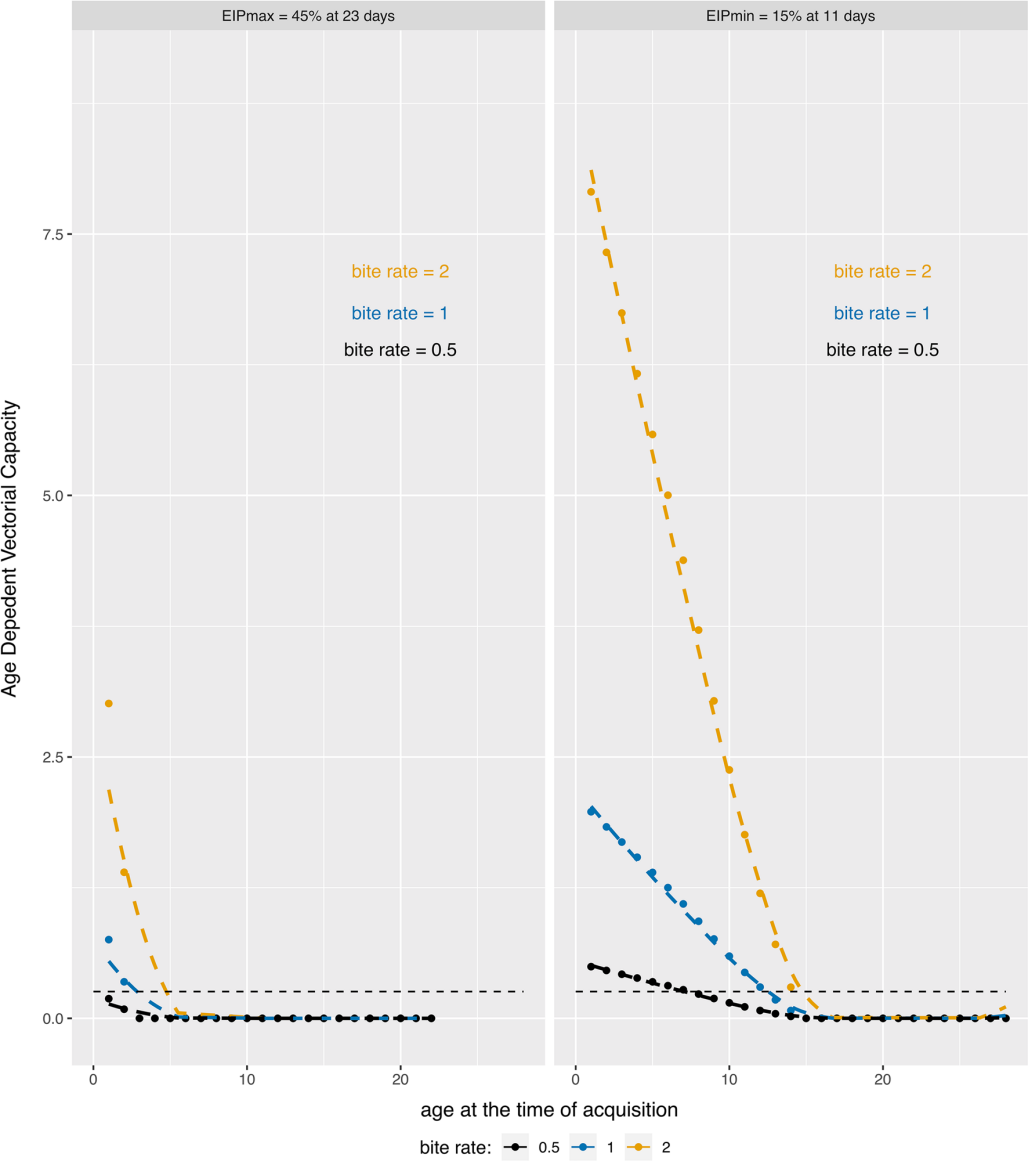
Vectorial capacity calculations using VC_age_. VC_age_ values (y-axis) depend on the age at acquisition (x-axis). Each curve calculated based on pre-determined bite rates of 2 (yellow line), 1 (blue line), and 0.5 (black line) for (left) EIP_max_ of the YOUNG group, = 45% at 23 dpi, and (right) EIP_min_ of the YOUNG group = 15% at 11 dpi. The dotted line represents the lower limit of VC where below this threshold, R_0_ is less than 1

To achieve an R_0_ of 1, the vectorial capacity needed to be at or above 0.256. When parameterized with EIP_max_, the VC_age_ at a biting rate of 0.5 (once every 2 days) did not achieve this minimal value, but at biting rates of once or twice per day, VC_age_ was sufficient to push R_0_ over the threshold of one (Fig. 5). We next determined the window of opportunity, which encompasses the days post-emergence when a mosquito acquires an infection and which results in VC_age_ values where R_0_ would be greater than one. The window of opportunity when we parameterized VC_age_ with EIP_max_ indicated that a virus must be acquired within the first 2 days post-emergence when the bite rate was twice or once per day (Fig. 5). Interestingly, when parameterized with EIP_min_, all bite rates reached sufficient VC_age_ values. The window of opportunity was 14 days post-emergence for a bite rate of twice a day, 12 days post-emergence for a bite rate of once a day, and 7 days post-emergence for a bite rate of once every 2 days (Fig. 5). Even at its highest, VC_age_ indicates that traditional VC calculations are likely overestimates.

### OLDER and S.OLDER groups

For both the OLDER and S.OLDER groups, the infectious blood meal occurred at 12 days post-emergence, which was outside the window of opportunity for VC_age_ in all but one scenario (Additional file 1: Figure S4). Only at a bite rate of twice daily did the OLDER group achieve the minimum VC_age_ of 0.256 on or after Age_acquisition_ of 12 days-old, and only when parameterized with the EIP_min_. The window of opportunity for this scenario was days 12–13 post-emergence.

With these vector age-dependent traits, both EIP and vector competence would need to be altered significantly to achieve a VC_age_ ≥ 0.256 at 12 days post-emergence for other scenarios. For example, we consider a hypothetical EIP_max_ of EIP_50_ (the time it takes for 50% of exposed mosquitoes to transmit) and a hypothetical EIP_min_ of EIP_10_ to demonstrate how these group-specific mortality rates and the age structure of willingness to bite interact to drive vectorial capacity above the threshold in the context of Age_acquisition_ [13, 49, 54]. The S.OLDER group would need a maximum EIP_50_ of 12 dpi or 11 dpi for biting rates of twice and once daily, respectively, and 8 dpi for a biting rate of once every 2 days. For the OLDER group, the maximum EIP_50_ would need to be 13, 12, or 11 dpi for biting rates of twice or once daily, or once every 2 days, respectively. With the EIP_min_, similar maximum EIPs were noted of 12 and 9 dpi for biting rates of twice or once daily, respectively. For both the OLDER and S.OLDER groups, with a hypothetical EIP_min_ of 10%, VC_age_ never got above the threshold needed to get R_0_ at or above 1.

## Discussion

Prediction of vector-borne disease spread remains difficult, as transmission of vector-borne disease is a dynamic, multifaceted system. This includes life traits of the mosquito, environmental factors, and vector-virus interactions [14, 16, 23, 55, 56]. Here we demonstrate through a combination of experimental and computational methods that mosquito age at the time of pathogen acquisition is a powerful driver of transmission potential due, in large part, to the age-dependence of daily mortality and biting habits. Further, these drivers lead to differences in the estimates of R_0_.

Our study, which focused upon the age at the time of infectious blood meal, showed no significant impacts on the vector competence of colony *Ae. aegypti* for ZIKV. While a recent study did show there were significant effects of multiple blood meals on vector competence [57], we delivered the second blood meal at a much longer interval (7 days *versus* 3 dpi in [57]), which likely plays a role in this disparate finding and indicates that there may be age-dependence in this phenomenon as well. However, this hypothesis is outside the scope of the present study. In our mortality study, we did observe a modest difference in TTD. We also observed a very short TTD in mosquitoes only given sugar with no blood meal. We chose not to pursue these effects, as the differences in TTD were modest and the impacts of no blood meal have been previously observed.

The technology for determining the age structure of natural mosquito populations in the field is currently still in development. For example, a study using near-infrared spectroscopy was able to predict the age of female *Ae. aegypti* ± 2 days, indicating that determining the age structure of a mosquito population is possible, and that such technology could be refined for field studies [58, 59]. Further, mid-infrared spectroscopy had varying, but some promising results in determining the age structure of *Anopheles* mosquitoes [59, 60]. As these technologies are pursued and refined, there will be a need for ways to understand and quantify age-dependent interactions among vector competence, EIP, mosquito lifespan, and biting behavior [16, 61].

The methodologies in this study highlight the need to understand the quantitative interaction between vector competence, mosquito mortality, and age at time of infection acquisition. Thus, we anticipate that this methodology could be used to explore other important modifiers of vector competence and mosquito mortality and the interaction of the two, such as innate mosquito immunity response to infection, adult and larval microbiome, as well as other extrinsic factors known to affect these traits [2, 3, 5, 10–15]. Several recent studies have focused on environmental factors such as temperature, and found that temperature not only affects vector competence of many arboviruses, especially in *Aedes aegypti*, but also several life traits of the mosquito [41, 55, 56]. This means that transmission is ultimately impacted by the interactions of all of these temporally dependent processes, and future studies should consider the age structure when assessing these impacts. Studies have also shown the impact of vector species, in particular the difference between field-derived and laboratory strains [2, 3]. Here, we used a laboratory strain of *Ae. aegypti*, which has been shown to vary in its competence when compared to a field-derived strain. Future use of VC_age_ could highlight these differences, as well as differences among various arbovirus-arthropod systems.

Our results demonstrate the importance of age structure when evaluating the fitness of a mosquito-virus system and indicate that R_0_ may be overestimated when it is not considered. This framework further shows that how within-mosquito arbovirus fitness is measured, often by comparing proportions of transmitting mosquitoes at arbitrary time points, is not sufficient. Here, when the Age_acquisition_ was advanced, the difference in hypothetical EIPs necessary for the system to succeed was not very different. For example, in the OLDER and S.OLDER groups, there was only one day difference between the EIP_min_ and EIP_max_ needed for success at biting rates of 2 and 1, though these quantities represented a difference of 40% in the proportion of mosquitoes transmitting. The same trend was demonstrated in the YOUNG group where EIP_min_ resulted in longer windows of opportunity compared to EIP_max_. Additionally, when the minimum time to transmission is shorter, a larger portion of the mosquito population contributes to transmission, because VC_age_ implicates older mosquitoes in this scenario given no significant reduction in mortality due to extrinsic factors [62]. Thus, VC_age_ reveals that the temporality of the within-mosquito arbovirus dynamics is more impactful than the ultimate magnitude of this widely used fitness measure. More investigations into the earlier and minimal transmission rates, such as at earlier time points that we did not consider, may be warranted to quantify fully the contribution of lower but faster dissemination profiles in arbovirus systems.

A study by Althouse et al. [63] also found that the temporality of transmission from non-human primates was sometimes more impactful than the magnitude of the viremia leading to transmission to the mosquito. They proposed a “tortoise-and-the-hare” (TatH) model to describe this relationship between arboviral viremia profiles in non-human primates and the predicted transmission success to vectors, showing that the strategy of “slow and steady” viremia, i.e. lower levels for longer periods, resulted in higher predicted transmission success of arboviruses [63]. This same TatH model recently described macro-transmission dynamics in Colombia, where it was demonstrated that slow burn-in epidemics actually resulted in cumulatively more cases and higher R_0_ values than in initially explosive outbreaks [64]. Vector competence is a continuous process over time, and as such, EIP_min_ and EIP_max_ are not independent measures. Thus, vector competence profiles with higher transmission rates at earlier times (“hare strategy”) will always be more fit than lower proportions at the same EIP. However, this study demonstrates that in systems where the maximum measured vector competence is low, but the time to minimum transmission is short (“tortoise strategy”) [65], there is still a good chance the system will succeed. VC_age_ also suggests that how arbovirus phenotypes are compared and ranked, and perhaps even how the field identifies ‘highly’ or ‘negligibly’ competent vectors, may need adjustment in the context of mosquito age.

## Conclusions

We demonstrate that mosquito age may not affect experimentally determined infection, dissemination, or transmission rates when assessed in the traditional days post-infection manner. However, when we consider mosquito mortality, EIP, and vector competence in the context of a more holistic measure of transmission potential, vectorial capacity, and further adjust VC for the effect of age at the time of mosquito infection, we demonstrate that age can be an important factor, and that how some arbovirus fitness measures are assessed may need reconsideration.

## Supplementary information

**Additional file 1: Figure S1.** Illustration of main treatment design for vector competence experimentation. **Table S1.** Infection and dissemination rates for each day post-infection (dpi) and corresponding mosquito age for each of the three ZIKV treatments. **Table S2.** Modeled fits of parameters, the type of model, and the parameter values. **Figure S2.** Observed and predicted daily probabilities of survival for the three treatment groups. **Text S1.** R packages used in the study. **Text S2.** Comparison of mortality of *Aedes aegypti* with respect to bloodmeals. **Figure S3.** Mortality curves for all treatments and associated controls. **Table S3.** Mean time to death and sample size for ZIKV-infection treatments and unexposed controls used in the mortality study. **Figure S4.** Demonstration of the VC_age_ for the two treatments exposed at older ages.

## Data Availability

All data generated or analyzed during this study are included in this published article and its additional file.

## Abbreviations

EIP: extrinsic incubation period
ZIKV: Zika virus
VC: vectorial capacity
dpe: days post-emergence
dpi: days post-infection
