# Supplementary material for: Age-structured vectorial capacity reveals timing, not magnitude of within-mosquito dynamics is critical for arbovirus fitness assessment
Source: Parasit Vectors. 2020 Jun 15;13:310. doi: 10.1186/s13071-020-04181-4 (PMC7296759; doi:10.1186/s13071-020-04181-4)
Supplement: Supplementary file 1 — Additional file 1: Figure S1. Illustration of main treatment design for vector competence experimentation. Table S1. Infection and dissemination rates for each day post-infection (dpi) and corresponding mosquito age for each of the three ZIKV treatments. Table S2. Modeled fits of parameters, the type of model, and the parameter values. Figure S2. Observed and predicted daily probabilities of survival for the three treatment groups. Text S1. R packages used in the study. Text S2. Comparison of mortality of Aedes aegypti with respect to bloodmeals. Figure S3. Mortality curves for all treatments and associated controls. Table S3. Mean time to death and sample size for ZIKV-infection treatments and unexposed controls used in the mortality study. Figure S4. Demonstration of the VCage for the two treatments exposed at older ages. [file 13071_2020_4181_MOESM1_ESM.pdf]

Additional file 1

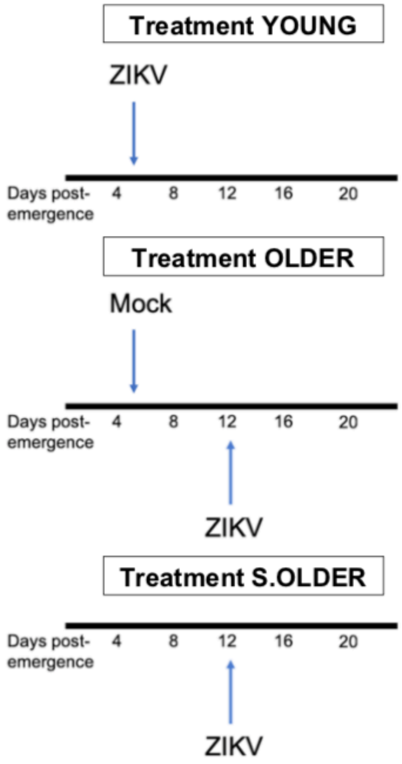

Figure S1. Illustration of main treatment design for vector competence experimentation. Three treatments were applied to assess our hypothesis. Treatment YOUNG received an infectious bloodmeal at 5 days old; Treatment OLDER received a mock (blood and non-infectious supernatant) bloodmeal at 5 days old, followed by an infectious bloodmeal at 12 days old; and Treatment S.OLDER (Sugar.OLDER) received an infectious bloodmeal at 12 dpe (no bloodmeal at 5 dpe).

Table S1. Infection and dissemination rates for each day post-infection (dpi) and corresponding mosquito age for each of the three ZIKV treatments. Percent infection was determined by the proportion of infected abdomens over total exposed; and percent dissemination was determined as the proportion of infected legs over total exposed. Three replicates were performed for each treatment, but proportions and sample sizes (n) are combined from all three replicates.

Treatment	dpi (Age)	% Infected (n)	% Disseminated (n)
YOUNG	5 (10)	77.45 (52)	38.53 (52)
	8 (13)	81.90 (50)	48.25 (50)
	11 (16)	83.38 (52)	69.93 (52)
OLDER	5 (17)	74.67 (45)	4.00 (45)
	8 (20)	69.40 (32)	37.50 (32)
	11 (23)	87.88 (44)	66.67 (44)
S.OLDER	5 (17)	78.27 (41)	9.80 (41)
	8 (20)	80.89 (50)	35.30 (50)
	11 (23)	74.09 (73)	51.94 (73)

Table S2. Modeled fits of parameters, the type of model, and the parameter values. For each parameter where predictions were made from experimental data, the type of model (and R self-starting function when applicable) and parameter estimates for each fit are given below. Goodness of fit was assessed either through AIC (for non-linear models) or R^2 for linear models.

Parameter	Model (R self-start function)	Parameter estimates	Goodness of fit
Daily probability of survival for YOUNG group	Asymptotic regression (SSasymp)	s = 0.796<br>r = 1.13<br>c = -2.56	AIC = -43.8
Daily probability of survival for OLDER group	Linear model	slope = -0.016<br>intercept = 1.13	Adj. R^2 = .88
Daily probability of survival for S.OLDER group	Linear model	slope = -0.012<br>intercept = 1.04	Adj. R^2 = .95
Probability of daily biting	Asymptotic regression (SSasymp)	s = 0.996<br>r = 0.995<br>c = -1.665	AIC = -0.71

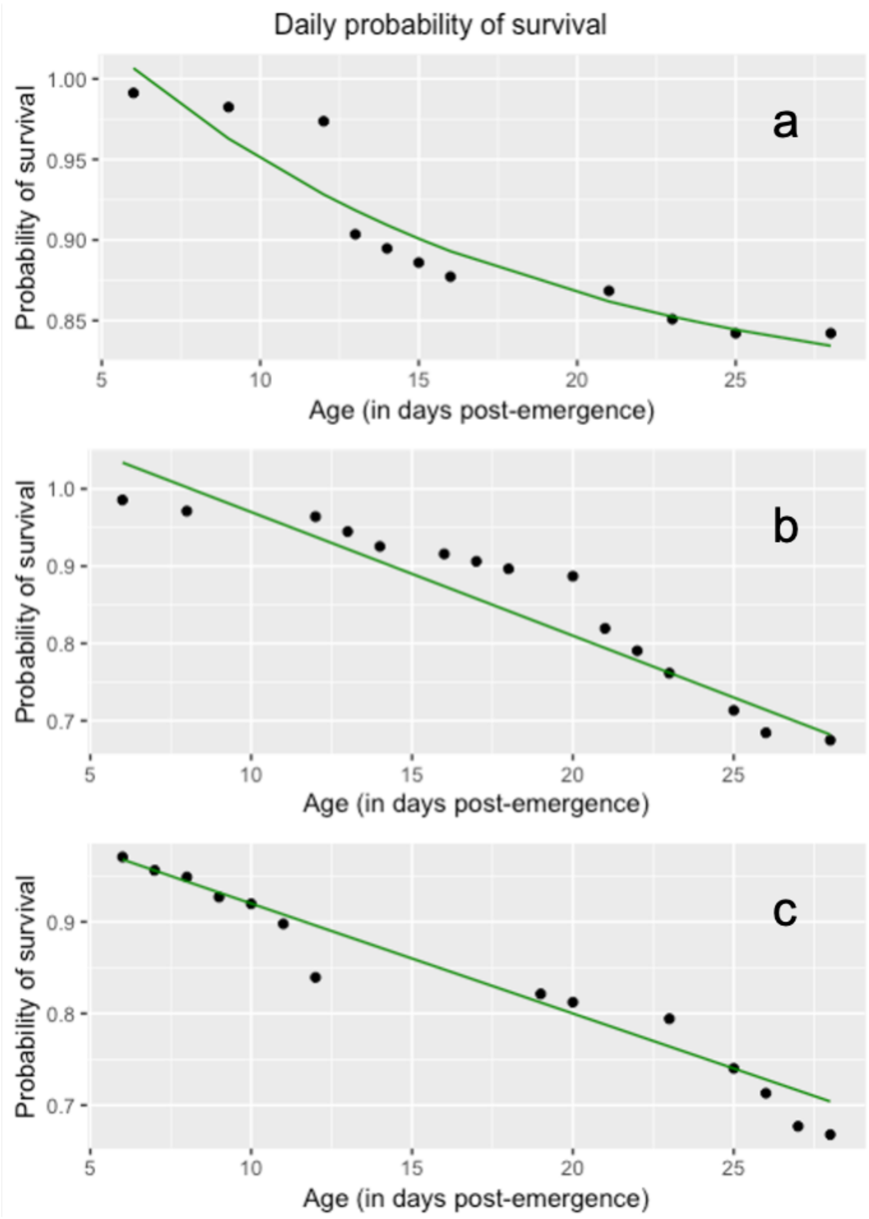

Figure S2. Observed and predicted daily probabilities of survival for the three treatment groups. Observed daily survival rates (dots) and the predicted daily survival rates (green curve) for YOUNG group (a), OLDER group (b), and S.OLDER group (c).

Text S1: R packages used in the study.

The R packages used in this study are as follows: ggplot2,ggthemes,gridExtra, doBy, MASS, stats, nlme, nls2, ggfortify, survival, ggpubr, ggplotify, lattice, vcd, reshape.

Text S2: Comparisons of mortality of *Aedes aegypti* with respect to bloodmeals
The supporting controls for each of the treatment groups were as follows:

1. For YOUNG group: a mock bloodmeal at 5 days post emergence (dpe) followed by sugar sustenance (“M.S”)
2. For OLDER group: a mock bloodmeal at 5 dpe followed by a second mock bloodmeal at 12 dpe (“M.M”)
3. For S.OLDER group: a mock bloodmeal at 12 dpe only (“S.M”)
4. A sugar-only control (“S”) that received no bloodmeals.

Of interest, the non-blood fed sugar-only controls (group S) died significantly faster than any of the other treatments with an average TTD of 19.6 days (Figure S3). To determine if there was a generalized effect of exposure on mortality, we compared all groups with a ZIKV exposure to those without, excepting the group that received no bloodmeal at all which was removed from the analysis. There was no significant difference between ZIKV-exposed mosquitoes (groups YOUNG, OLDER, and S.OLDER) and the non-exposed groups ($p>0.05$).

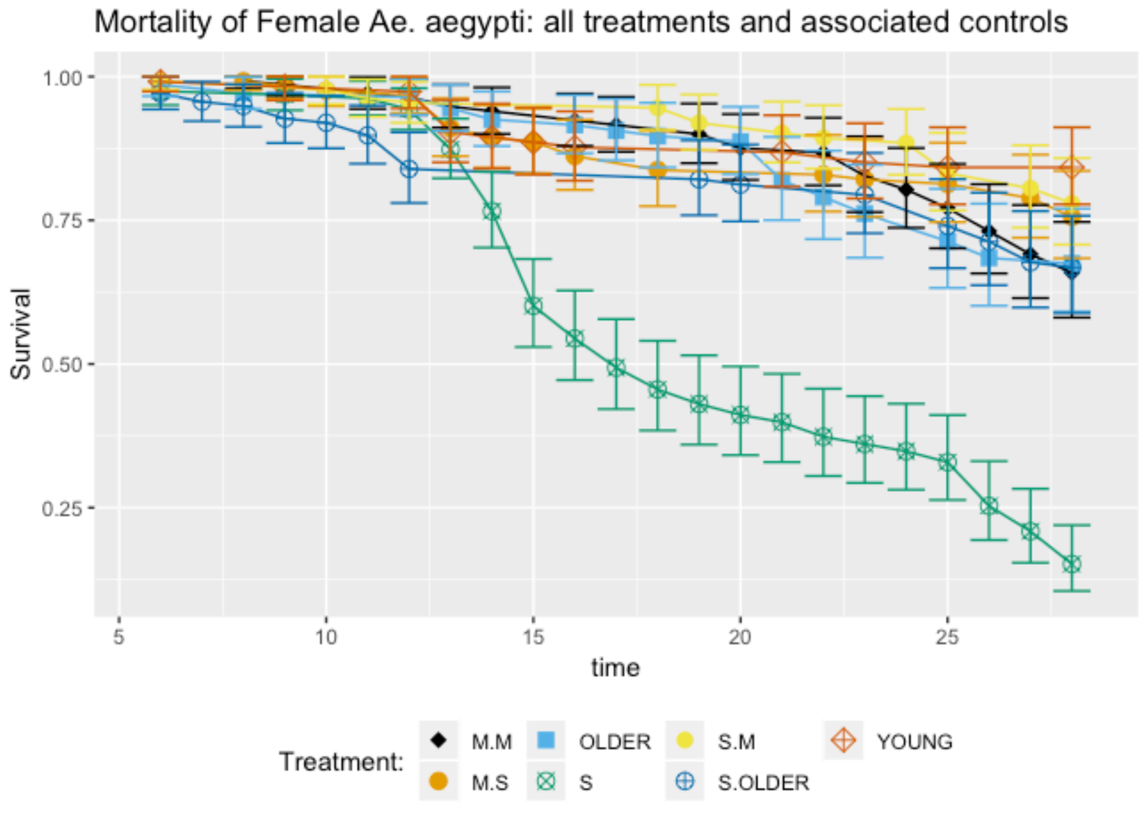

Figure S3: Mortality curves for all treatments and associated controls. The sugar-only group had significantly faster mortality compared to the other groups which received at least one bloodmeal.

Table S3. Mean time to death and sample size for ZIKV-infection treatments and unexposed controls used in the mortality study. Exposed groups were the three treatments exposed to ZIKV: YOUNG -- ZIKV at 5 days post emergence (dpe); OLDER – mock bloodmeal at 5 dpe and ZIKV bloodmeal at 12 dpe; S.OLDER – only a ZIKV bloodmeal at 12 dpe. Unexposed groups were not exposed to ZIKV but matched for bloodmeal uptake: M.S – mock bloodmeal at 5 dpe; M.M – mock bloodmeals at 5 and 12 dpe; S.M – mock bloodmeal at 12 dpe; and S – no bloodmeal, sugar only. Mean times to death and total sample sizes per treatment are given below.

**Group**	**Treatment**	**Time to death**	**Sample size (n)**
Exposed	YOUNG	25.9	114
	OLDER	25.3	139
	S.OLDER	24.5	137
Unexposed	M.S	25.5	123
	M.M	25.8	139
	S.M	26.5	132
Sugar Only	S	19.6	158

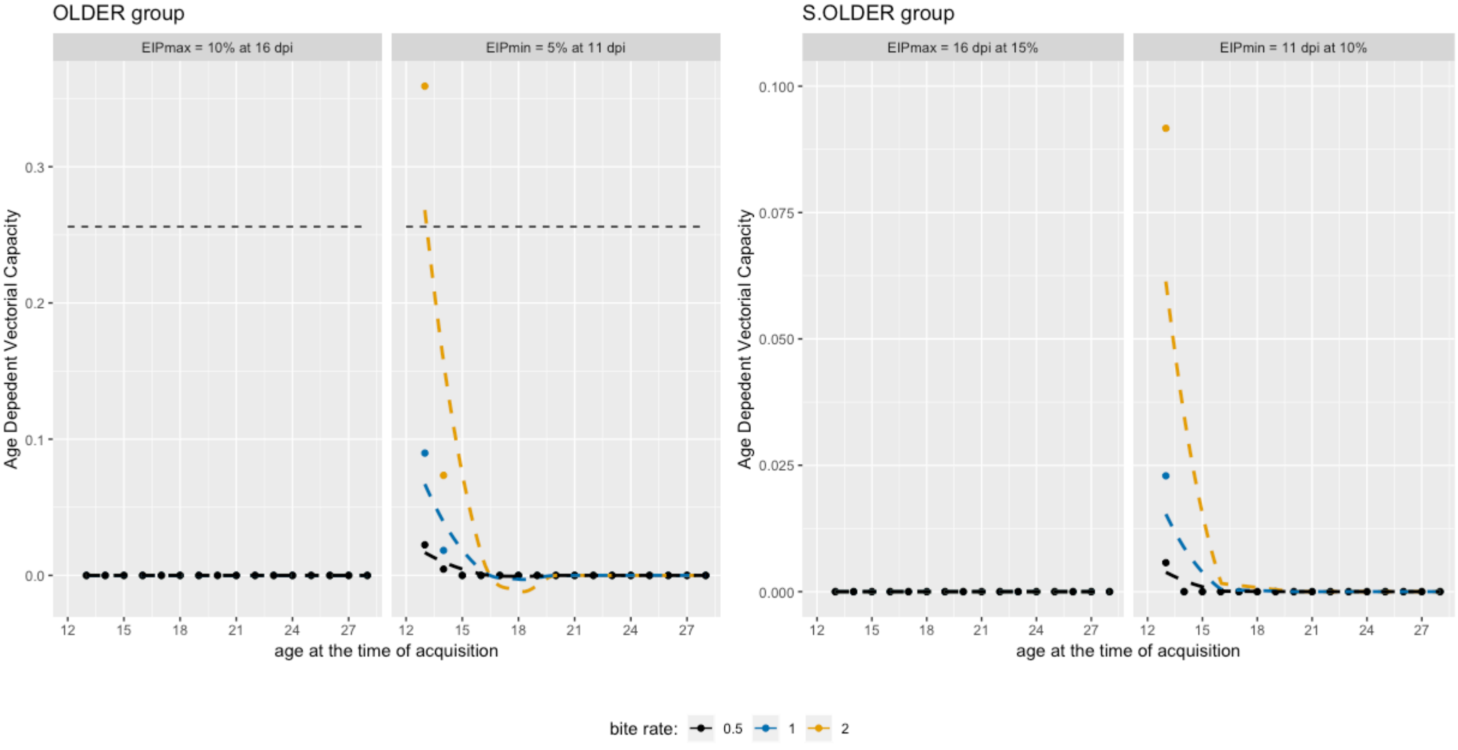

Figure S4. Demonstration of the VC_{age} for the two treatments exposed at older ages. VC_{age} values for the OLDER and S.OLDER groups indicates that $Age_{acquisition}$ of 12 days post emergence was too late in all but one case to result in $R_0 \geq 1$ in this model system. The dotted line is where VC_{age} = 0.256 (where applicable).
